# Akt inhibition attenuates rasfonin-induced autophagy and apoptosis through the glycolytic pathway in renal cancer cells

**DOI:** 10.1038/cddis.2015.344

**Published:** 2015-12-03

**Authors:** Q Lu, S Yan, H Sun, W Wang, Y Li, X Yang, X Jiang, Y Che, Z Xi

**Affiliations:** 1 State Key Laboratory of Mycology, Institute of Microbiology, Chinese Academy of Sciences, Beijing, China; 2 University of Chinese Academy of Sciences, Beijing, China; 3 State Key Laboratory of Toxicology and Medical Countermeasures, Beijing Institute of Pharmacology and Toxicology, AMMS, Beijing, China; 4 Department of Urology, Peking University First Hospital, Beijing, China

## Abstract

Rasfonin is a fungal secondary metabolite with demonstrated antitumor effects. However, the underlying mechanism of the regulatory role in autophagy initiated by rasfonin is largely unknown. Moreover, the function of Akt to positively mediate the induced autophagy remains elusive. In the present study, we observed that rasfonin induced autophagy concomitant with the upregulation of Akt phosphorylation. Both the inhibition of Akt by small molecule inhibitors and genetic modification partially reduced rasfonin-dependent autophagic flux and PARP-1 cleavage. The overexpression of myrAkts (constant active form) promoted rasfonin-induced apoptosis and autophagy in a cell type- and Akt isoform-specific manner. Using quantitative PCR and immunoblotting, we observed that rasfonin increased the expression of glycolytic gene *PFKFB3*, and this increased expression can be suppressed in the presence of Akt inhibitor. The inhibition of PFKFB3 suppressed rasfonin-activated autophagy with enhanced PARP-1 cleavage. In the case of glucose uptake was disrupted, which mean the glycolytic pathway was fully blocked, the rasfonin-induced autophagy and PARP-1 cleavage were downregulated. Collectively, these results demonstrated that Akt positively regulated rasfonin-enhanced autophagy and caspase-dependent apoptosis primarily through affecting the glycolytic pathway.

On the basis of distinct cell morphology, three major types of cell death have been described: apoptosis, autophagic cell death, and programmed necrosis.^1, 2, 3^ Accumulating evidence suggests the existence of several molecular connections among apoptosis, necrosis, and autophagy.^3, 4^ Macroautophagy (hereafter called autophagy), an evolutionarily conserved catabolic and intracellular membrane trafficking process, is involved in the delivery of cytoplasmic contents and organelles to lysosomes for degradation.^5^ In general, the mammalian target of rapamycin (mTOR) is a negative regulator of autophagy.^6, 7, 8^ As a member of the PI3K-related kinase family, mTOR has been detected in two distinct complexes, mTORC1 and mTORC2, which regulate many aspects of cellular functions.^9, 10^ mTORC2 activates Akt (protein Kinase B), while PI3K/Akt primarily activates mTORC1.^11^ Once activated by Akt, mTORC1 elicits a negative feedback loop to inhibit the activity of Akt. mTORC1 phosphorylates two main substrates, ribosomal protein S6 kinase 1 (S6K1) and eukaryotic initiation factor 4E-binding protein 1 (4E-BP1).^12^

As an upstream regulator of mTOR, Akt is usually considered to be an autophagy suppressor, and the Akt inhibitor can be used as an autophagy inducer.^13^ Three highly homologous Akt isoforms (Akt1, Akt2, and Akt3), encoded by separate genes, are expressed in mammalian cells.^14^ Akt is perhaps the most frequently activated oncoprotein in human cancers, and its activation contributes to the genesis of cancer through the inhibition of apoptosis and induction of proliferation.^15^ However, a recent study suggested that Akt isoforms showed opposite functions in tumor initiation and growth.^16^ Moreover, the overexpression of constitutively active Akt isoforms inhibits the proliferation of MDA-MB-231 cells.^17^

Warburg effect, a hallmark of cancer, was first discovered by Otto Warburg.^18, 19^ In this process, cancer cells shift to glycolytic energy dependence with or without molecular oxygen. Akt activation increased the total cellular ATP content, whereas Akt deprivation reduced intracellular ATP levels.^20^ Growing evidence indicates that Akt has a major role in the coordinated regulation of both glycolytic and oxidative metabolism.^21^ Akt augments the glycolytic flux through several mechanisms, such as increasing the expression of glucose transporters, enhancing the coupling between oxidative phosphorylation and glycolysis, promoting the accumulation of HIF1α and HK2, and activating phosphofructokinase-2 (PFK-2).^18^ Here, ACHN cell line was selected as the experiment material, as renal cell carcinoma (RCC) is a model for the role of Warburg effect leading to malignancy.^22^

In mammals, several PFK-2/FBPase-2 isoenzymes are encoded by four different genes.^23^ These isoenzymes control glycolysis via the maintenance of the cellular levels of fructose-2,6-bisphosphate (F26BP), a major allosteric activator of 6-phosphofructo-1-kinase (PFK-1), a critical rate-limiting enzyme of glycolysis. A previous study reported that the knockdown of 6-phosphofructo-2-kinase/fructose-2, 6-bisphosphatase 3 (PFKFB3), a member of the PFK-2 family, suppressed autophagy.^24^ Given the intimate association between Akt and glycolysis, we speculated that Akt might regulate autophagy via the glycolytic pathway.

Rasfonin is a natural product isolated from the fermented mycelium of *Talaromyces* sp. 3656-A1, named according to the biological activity of this compound against the small G-protein Ras. Recently, rasfonin was shown to induce the death of ras-mutated pancreatic tumor (Panc-1) cells.^25^ In the present study, we demonstrated that rasfonin induces autophagy, which contributes to apoptosis. Moreover, this compound activates autophagy concomitant with the upregulation of Akt phosphorylation. API-2 and SC66, two inhibitors of Akt, attenuated both autophagy and caspase-dependent apoptosis concomitantly with an alteration in PFKFB3 expression. Although PFK-15 and 3-PO, two inhibitors of PFKFB3,^26^ decreased the magnitude of autophagy and increased the rasfonin-induced cleavage of PARP-1, the inhibition of glucose uptake by 2-Deoxyglucose (2-DG) or glucose-free medium reduces both rasfonin-dependent autophagy and apoptosis.

## Results

### Rasfonin inhibits cell viability and activates multiple cell death pathways in ACHN cells

**Figure 1:**
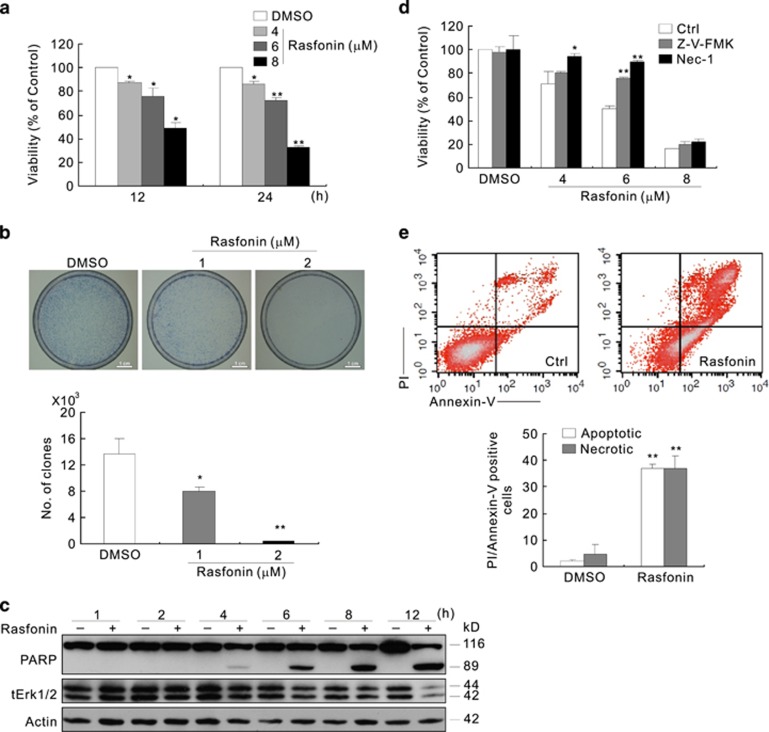
Rasfonin inhibits cell viability and activates multiple cell death pathways in ACHN cells. (**a**) ACHN cells were treated with rasfonin (4–8 *μ*M) for up to 24 h; cell viability was analyzed by MTS assay as described in Materials and Methods. Data are presented as mean±S.D., and are representatives of three independent experiments. Each performed in triplicate, and the data were analyzed by *T*-test. (**b**) Colony growth assays were performed in ACHN cells with rasfonin (1 and 2 *μ*M). (**c**) The cells were treated with rasfonin (6 *μ*M) upon to 12 h, and then cell lysates were prepared and analyzed by immunoblotting using the indicated antibodies; actin was used as a loading control. (**d**) ACHN cells were treated with rasfonin (6 *μ*M) for 24 h in the presence or absence of Z-V-FMK (20 *μ*M) or Nec-1 (30 *μ*M), cell viability was analyzed by MTS assay. Ctrl: cells with equal amount of DMSO. (**e**) Following treatment of the cells with rasfonin (6 *μ*M) for 12 h, the apoptosis and necrosis induced were determined by flow cytometry. Apoptotic: AV-positive and PI-negative; necrotic: PI-positive. The data are presented as mean±S.D. from three independent experiments. The single asterisk denotes the group is statistically different from the control groups (*P*<0.05), whereas double asterisk means *P*<0.01

In the present study, rasfonin-induced cell death was first detected using the human renal cancer cell line ACHN, and rasfonin reduced the viability of ACHN cells in a time- and dose-dependent manner (Figure 1a). These findings were confirmed by colony growth assay, in which rasfonin inhibited the cell growth depending on the concentration of stimulus (Figure 1b). Immunoblotting analysis showed that rasfonin induced cleavage of PARP-1 (Figure 1c), PARP-1 is one of the main cleavage targets of caspase-3 *in vivo*, and cleavage of PARP-1 serves as a marker of cells undergoing apoptosis,^27, 28^ suggesting the activation of caspase-dependent apoptotic pathway. As the pan-caspase inhibitor Z-V-FMK blocks caspase-dependent apoptosis,^29^ we examined rasfonin-dependent cell death in the presence of Z-V-FMK; and the results showed that the pre-treatment of ACHN with this inhibitor provided only partial protection against rasfonin-induced cell death (Figure 1d). Necrostatin 1 (Nec-1), an inhibitor of necroptosis,^30^ offered a greater protection of cell viability than that of Z-V-FMK in rasfonin-treated cells (Figure 1d), implying that rasfonin activated multiple cell death pathways in a dose-dependent manner. Additionally, flow cytometry data revealed that the rasfonin-induced cell death of ACHN could be either apoptotic or necrotic (Figure 1e).^31^

### The inhibition of autophagy partially rescues cell viability and attenuates rasfonin-induced PARP-1 cleavage

**Figure 2:**
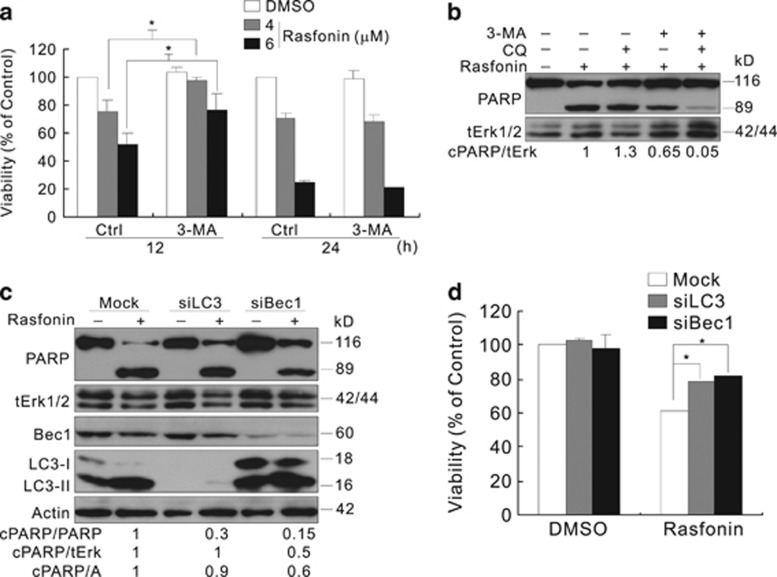
The inhibition of autophagy partially rescues cell viability and attenuates rasfonin-induced PARP-1 cleavage. (**a**) ACHN cells were treated with rasfonin (6 *μ*M) upon to 24 h in the presence or absence of 3-MA (2 mM), cell viability was analyzed by MTS assay. (**b**) Following treatment of the cells with rasfonin (6 *μ*M) for 12 h in the presence or absence of CQ (10 *μ*M) or 3-MA (2 mM), immunoblotting was carried out with the indicated antibodies; tERK1/2 was used as a loading control. (**c**) ACHN cells were transfected with siRNA target LC3 or Beclin1 (Bec1) for 48 h. Cell lysates were analyzed by immunoblotting with the indicated antibodies following 12 h rasfonin (6 *μ*M) treatment; actin was used as a loading control. Densitometry was performed for quantification and relative ratios of cleaved PARP-1 (cPARP-1) were shown below the blots. (**d**) Cell viability of cells from (**c**) was analyzed by MTS assay. The single asterisk denotes statistically different between the marked groups (*P*<0.05)

The widely used inhibitor of autophagy, 3-Methyladenine (3-MA),^32^ suppressed rasfonin-induced cell death and PARP-1 cleavage at the 12-h time point (Figures 2a and b). At the 24-h time point, 3-MA no longer provided protection for cell viability (Figure 2a), whereas, chloroquine (CQ), a known inhibitor of autophagosome-lysosome fusion,^8^ was found to increase the PARP-1 cleavage (Figure 2b). However, the combination of 3-MA and CQ further decreased rasonin-induced PARP-1 cleavage (Figure 2b). To confirm these results, we knocked down two important autophagy genes, Beclin1 (Bec1) or LC3.^8^ We observed that the elimination of Bec1 and LC3 expression inhibited the rasfonin-induced PARP-1 cleavage (Figure 2c), whereas the deprivation of either gene partially protected cell viability (Figure 2d). These findings indicated that autophagy is involved in rasfonin-induced caspase-dependent apoptosis.

### Rasfonin enhances autophagy with a concomitant downregulation of mTORC1 signaling and upregulation of Akt activity

**Figure 3:**
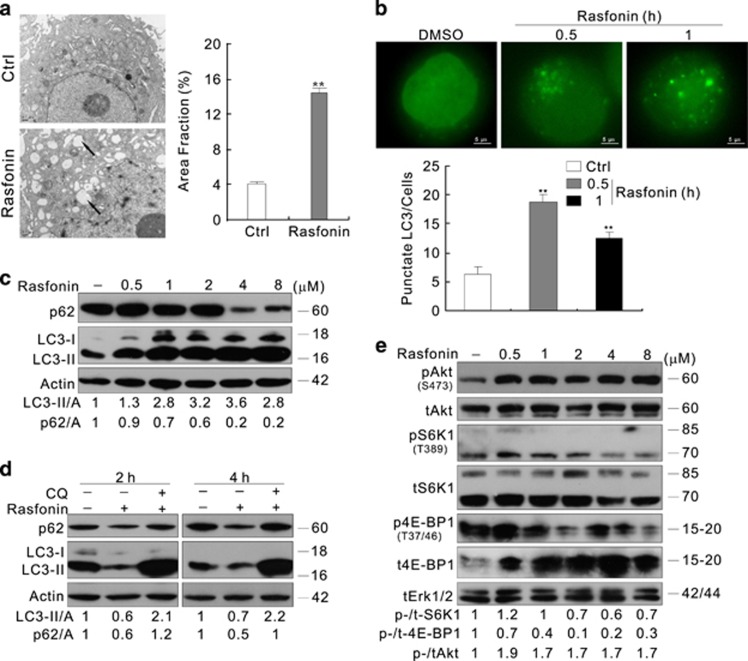
Rasfonin enhances autophagy. (**a**) Electron microscopy was performed in ACHN cells following treatment of rasfonin (6 *μ*M) for 1 h, the area indicated by the arrow represents the autophagosome. The morphometric analysis of the area fraction between autophagosomes and cytoplasm was calculated by using the Photoshop software. The data of the area ratio were non-normally distributed, and are presented as the mean of at least 10 cells counted for each group. (**b**) The cells were transfected with GFP-LC3 for 24 h. The cells were split onto coverslips and cultured overnight, 4% paraformaldehyde fixed, and visualized by confocal microscopy following rasfonin (6 *μ*M) for indicated time. The number of the punctate GFP-LC3 in each cell was counted, and at least 50 cells were included for each group. Data representing the mean±S.D. were shown in graph. (**c**–**e**) ACHN cells were treated with rasfonin (**d**: 6 *μ*M) upon to 4 h (**c** and **e**: 1 h) in the presence or absence of CQ (10 *μ*M). The cells were lysed and subjected to immunoblotting with the antibodies indicated. Densitometry was performed for quantification, and the adjusted ratio of LC3-II and p62 to actin (A), relative levels of phosphorylated Akt, 4E-BP1, and S6K1 were presented below the blots. tERK1/2 was used as a loading control in (**e**). Data represent three independent experiments. The double asterisk denotes statistically different between the marked groups (*P*<0.01)

Electron microscopy (EM), considered as one of the most convincing approaches to detect autophagy,^8^ was used to determine whether rasfonin enhances autophagy. Compared with the control, an obvious accumulation of membrane vacuoles was observed in rasfonin-treated ACHN cells (Figure 3a). The rasfonin-treated ACHN cells were transfected with a green fluorescent protein (GFP) and LC3 fusion protein and subsequently observed using fluorescence or confocal microscopy.^33^ Similar to the EM results, rasfonin rapidly increased the punctate staining of GFP-LC3 at both the 0.5- and 1-h time points (Figure 3b). The immunoblotting analysis revealed that rasfonin treatment increased the ratio of LC3-II to actin relative to control cells in a concentration-dependent manner (Figure 3c). Moreover, we observed that p62/SQSTM1, a selective substrate of autophagy,^8^ decreased in rasfonin-exposed cells (Figure 3c). To detect autophagic flux, the level of LC3-II was measured in the presence of CQ. As expected, CQ addition resulted in further accumulation of LC3-II in ACHN cells (Figure 3d), indicating that rasfonin induces autophagy. Although rasfonin decreased LC3-II levels at the 2- and 4-h time points (Figure 3d), CQ induced LC3-II accumulation in rasfonin-treated cells (Figure 3d). At the same time, p62 was decreased following rasfonin treatment, and CQ blocked this progress, suggesting that autophagic flux is enhanced under these conditions. Using quantitative RT-PCR (qRT-PCR), we observed that rasfonin showed no sign of the inhibition of either LC3 or p62 expression (Supplementary Figure 1A), indicating that the rasfonin-induced degradation of p62 and LC3-II is a post-transcriptional event. Similarly results were also obtained in another renal cancer cell line 786-O (Supplementary Figure 1B and C).

Generally, mTOR inhibits autophagy, and the kinase activity of mTOR is inferred by measuring the phosphorylation of substrates, such as S6K1 and 4E-BP1.^6, 8^ To determine whether rasfonin-induced autophagy is associated with mTOR pathway, the phosphorylation of S6K1 and 4E-BP1 was examined; and the results showed that the phosphorylation of these proteins decreased in a concentration-dependent manner in response to rasfonin in ACHN cells (Figure 3e). In addition, we observed that rasfonin activated autophagy concomitantly with the upregulation of Akt activity (Figure 3e).

### Akt inhibition suppresses rasfonin-induced autophagy and PARP-1 cleavage

**Figure 4:**
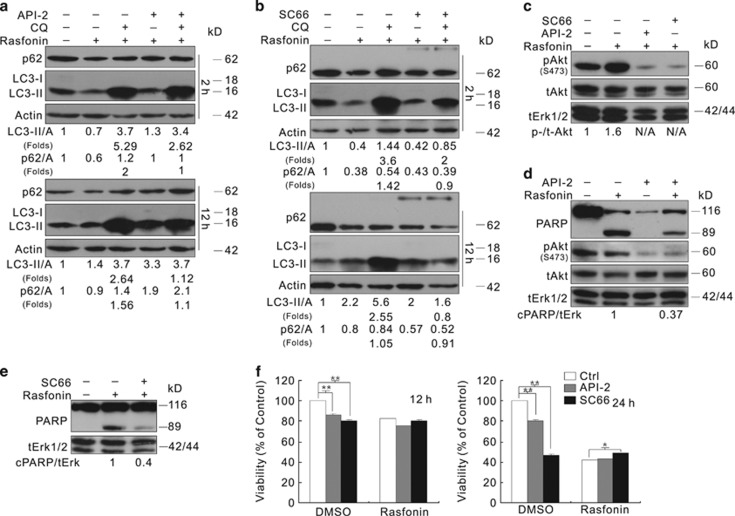
Akt inhibition diminishes Rasfonin-induced autophagic process and PARP-1 cleavage. ACHN cells were treated with rasfonin (6 *μ*M) or together with API-2 (2.5 *μ*M), or SC66 (8 *μ*M) upon to 12 h ((**a**) and (**b**): 2 h and 12 h; (**c**): 2 h; (**d**) and (**e**): 12 h) in the presence or absence of CQ (10 *μ*M), and cells were lysed and subjected to immunoblotting with the indicated antibodies. Densitometry was performed for quantification, and relative levels of LC3-II, p62, and cPARP-1 were presented below the blots. Similar experiments were repeated three times. N/A, not available. (**f**) ACHN cells were treated with rasfonin (6 *μ*M) upon to 24 h in the presence or absence of API-2 (2.5 *μ*M) or SC66 (8 *μ*M), cell viability was analyzed by MTS assay. tERK1/2 was used as a loading control in (**c**–**e**). The single asterisk denotes statistically different between the marked groups (*P*<0.05), whereas double asterisk means *P*<0.01

API-2 has been previously demonstrated to stimulate autophagy.^13^ We also observed that API-2 induced and enhanced Rapa-dependent autophagy in ACHN cells (Supplementary Figure 2A and B). In contrast, the combination of rasfonin with API-2 did not stimulate autophagy at the 1-h time point (Supplementary Figure 2C), as the addition of CQ failed to induce LC3-II accumulation.^8, 34^ Compared with rasfonin treatment alone, the combination of rasfonin with API-2 accumulated less LC3-II in the presence of CQ and decreased p62 degradation at both the 2- and 12-h time points (Figures 4a and c). To confirm these results, we conducted experiments using another Akt inhibitor, SC66.^35^ The induced autophagy was reduced upon SC66 challenge (Figures 4b and c), and the higher molecular mass of p62 was detected in SC66-treated cells (Figure 4b). In addition, both API-2 and SC66 suppressed rasfonin-induced cleavage of PARP-1 (Figures 4d and e). Consistent with the results obtained in ACHN cells, we observed either API-2 or SC66 suppressed rasfonin-induced autophagy and PARP-1 cleavage in 786-O cells (Supplementary Figure 2D and E). Only the combination of rasfonin with API-2, but not SC66, showed synergistic inhibition on cell viability only at 24-h time point (Figure 4f). Moreover, we observed that either API-2 or SC66 suppressed cell viability (Figure 4f), whereas SC66 showed a greater inhibitory effect on cell viability than API-2.

### Overexpression of activated Akt regulates the rasfonin-dependent autophagy in a time- and cell type-dependent manner

**Figure 5:**
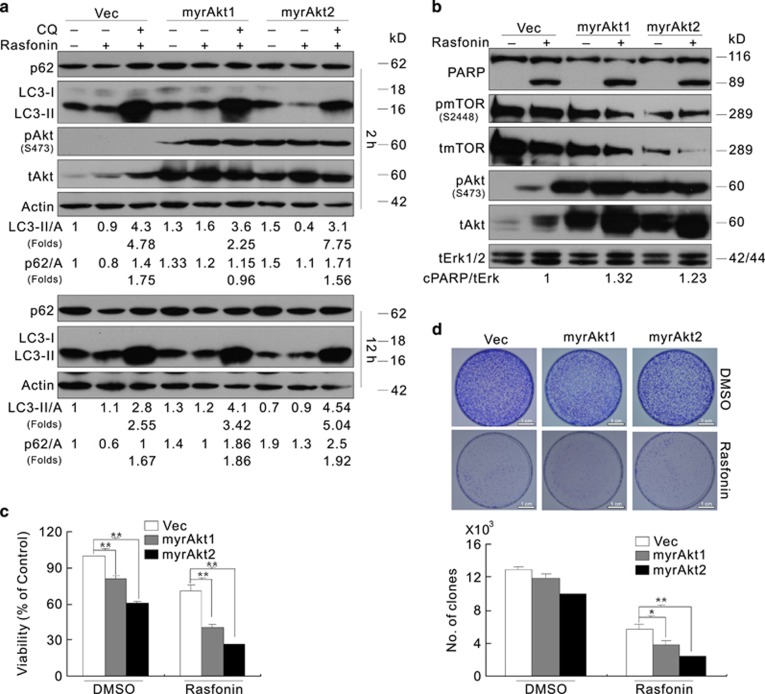
The overexpression of myrAkt affects rasfonin-dependent autophagy in an Akt isoform-specific manner. ACHN cells were transfected with the indicated plasmids for 36 h. (**a** and **b**) Following treatment of rasfonin (6 *μ*M) upon to 12 h (**a**: 2 h; **b**: 12 h) in the presence or absence of CQ (10 *μ*M), the lysates were analyzed by immunoblotting with the indicated antibodies. Densitometry was performed for quantification, and relative levels of LC3-II, p62, and cPARP-1 were presented below the blots; tERK1/2 was used as a loading control in (**b**). (**c**) Cell viability was analyzed by MTS assay following treatment of rasfonin (6 *μ*M) for 24 h. (**d**) Colony growth assays were performed in vector- (Vec) or myrAkt-transfected ACHN cells with rasfonin (2 *μ*M). Similar experiments repeated twice. The single asterisk denotes statistically different between the marked groups (*P*<0.05), whereas double asterisk means *P*<0.01

The expression of myrAkt1 inhibits autophagy in HeLa cells.^36^ Consistently, here, we demonstrated that the overexpression of either myrAkt1 or myrAkt2 suppressed rasfonin-induced autophagy in these cells (Supplementary Figure 3A). However, rasfonin-dependent autophagy was inhibited through the overexperession of myrAkt1, but not myrakt2, in ACHN cells at the 2-h time point, evidenced as LC3-II accumulation in the presence of CQ and p62 degradation (Figure 5a). Moreover, rasfonin and CQ increased LC3-II accumulation in both myrAkt1- and myrAkt2-transfected ACHN cells at the 12-h time point (Figure 5a). In addition, the overexpression of myrAkt1 did not inhibit the induced autophagy at 1- and 4-h time points (Supplementary Figure 3B). These observations suggest that Akt isoforms may function differentially in autophagy regulation depending on stimulation duration and cell type.

In addition, the overexpression of activated Akt increased PARP-1 cleavage in response to rasfonin challenge (Figure 5b), and showed no protection for cell viability (Figure 5c). The results of the colony assay revealed that exogenous activated Akt did not prevent rasfonin-mediated cell death (Figure 5d). Indeed, the expression of either myrAkt1 or myrAkt2 alone reduced cell viability (Figure 5c). Moreover, activated Akt decreased the phosphorylation of mTOR (Figure 5b). Thus, myrAkts might stimulate PARP-1 cleavage via the downregulation of mTOR signaling. To examine this hypothesis, mTOR was knocked down in ACHN cells, and the reduction of mTOR expression increased both Akt phosphorylation and rasfonin-induced PARP-1 cleavage (Supplementary Figure 3C).

### Knockdown of Akt reduces the magnitude of autophagy induction in ACHN cells

**Figure 6:**
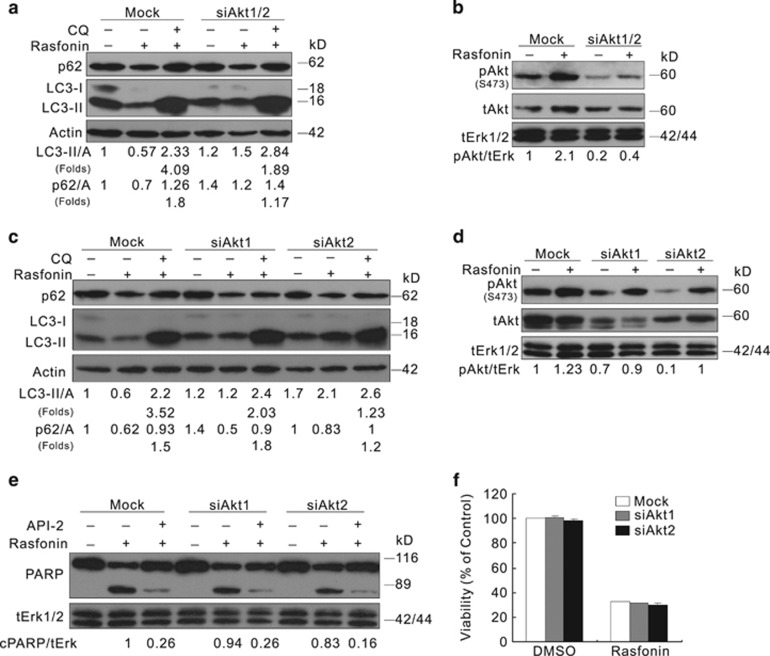
Akt deprivation lessens the induced autophagic flux. (**a**–**e**) ACHN cells were transfected with the indicated siRNAs for 48 h. The lysates were analyzed by immunoblotting following rasfonin (6 *μ*M) for 2 h (**a**–**d**) or 12 h (**e**) in the presence or absence of CQ (10 *μ*M). (**f**) Cell viability was analyzed by MTS assay following treatment of rasfonin (6 *μ*M) for 24 h. Relative levels of LC3-II, p62, and cPARP-1 were calculated and presented below the blots. tERK1/2 was used as a loading control in (**b**, **d** and **e**). Similar experiments repeated three times

To further examine the role of Akt in rasfonin-dependent autophagy, small RNA interference (siRNA) was employed to ablate Akt1/2. Akt1/2 silencing decreased rasfonin-induced accumulation of LC3-II in the presence of CQ and p62 degradation (Figures 6a and b). To explore the isoform-specific roles of Akt in autophagy, each individual Akt isoform was ablated, and Akt2 ablation resulted in a more significant increase in the inhibition of autophagy compared with Akt1 depletion (Figures 6c and d). Therefore, Akt isoforms exert unequal regulatory roles in the induced autophagy.

As to the apoptosis, Akt1 or Akt2 silencing only partly decreased the rasfonin-activated PARP-1 cleavage (Figure 6e). This phenomenon may be due to the complementary effect of other Akt isoforms. As the addition of API-2 further decreased the rasfonin-induced PARP-1 cleavage, and nearly blunted the difference between the Akt isoform-specific knockdown cells and control cells (Figure 6e). In 786-O cells, the expression status of Akt1 affected the rasfonin-induced PARP-1 cleavage similarly to the results observed in ACHN cells (Supplementary Figure 3D and E). Moreover, the deprivation of either Akt1 or Akt2 had no effect on the protection of cell viability in response to rasfonin challenge in ACHN cells (Figure 6f).

### Akt inhibition reduces the expression of glycolytic genes

Akt augments glycolytic flux through several mechanisms.^18, 37^ Recent studies suggested that PFKFB3 is involved in the regulation of autophagy.^24, 38^ Thus, we speculated that Akt might positively regulate rasfonin-induced autophagy by affecting the glycolytic pathway. Using qRT-PCR, we revealed that rasfonin increased the mRNA expression of PFKFB3 in response to stimulation for up to 8 h (Supplementary Figure 4A), and either API-2 or SC66 reduced gene expression with or without the agent treatment (Supplementary Figure 4B). Consistently, rasfonin increased the expression of PFKFB3 at protein level (Supplementary Figure 4C). Although activated Akt1 have no effect on the expression of PFKFB3 (Supplementary Figure 4D), Akt1 deprivation and API-2 treatment decreased PFKFB3 levels (Supplementary Figure 4E and F). Notably, rasfonin increased the expression of PFKFB3 in vector-transfected, but not in Mock-control cells (Supplementary Figure 4D and E), suggesting that rasfonin affects the glycolytic pathway in a context-dependent manner.

### Inhibition of PFKFB3 suppresses rasfonin-induced autophagy

**Figure 7:**
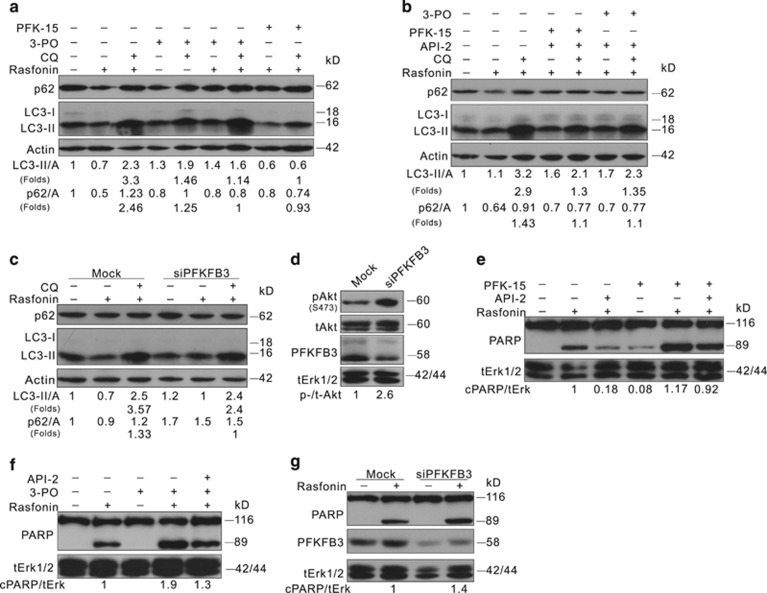
Inhibition of PFKFB3 suppresses rasfonin-induced autophagic process, whereas fails to decrease rasfonin-induced PARP-1 cleavage. (**a**, **b**, **e**, and **f**) ACHN cells were treated with rasfonin (6 *μ*M) or together with API-2 (2.5 *μ*M), PFK-15 (6 *μ*M), or 3-PO (10 mM), or with the indicated compounds in the presence or absence of CQ (10 *μ*M) for 2 h (**a** and **b**) or 12 h (**e** and **f**). (**c**, **d**, and **g**) ACHN cells were transfected with the indicated siRNA for 48 h. Following treatment of rasfonin (6 *μ*M) in the presence or absence of CQ (10 *μ*M) upon to 12 h (**c** and **d**: 2 h; **g**: 12 h). Cell lysates were analyzed by immunoblotting with the antibodies indicated. Relative levels of LC3-II, p62, and cPARP-1 were calculated and presented below the blots. tERK1/2 was used as a loading control in (**d**–**g**). Similar experiments repeated twice

PFK-15, an inhibitor of PFKFB3, was found to decrease LC3-II at 2-h time point (Supplementary Figure 5A). Additionally, PFK-15 attenuated the glycolysis pathway as the secreted lactate was significantly decreased (Supplementary Figure 5B). Although 3-PO, another inhibitor of PFKFB3, accumulated LC3-II in the presence of CQ, the combination of 3-PO with CQ did not augment p62 (Figure 7a). Furthermore, both PFK-15 and 3-PO exerted an inhibitory effect on rasfonin-induced autophagy (Figure 7a), as CQ failed to induce LC3-II accumulation. Interestingly, although PFK-15 or 3-PO alone increased Akt phosphorylation, this agent did not further activate Akt upon rasfonin stimulation (Supplementary Figure 5C and D). Then, cells treated with a combination of API-2 and rasfonin were further stimulated with PFKFB3 inhibitor PFK-15, showing that the presence of both API-2 and PFK-15 completely or markedly inhibited rasfonin-dependent autophagy at 2-h time point (Figure 7b). Notably, another inhibitor of PFKFB3, 3-PO, showed a similar effect (Figure 7b). Consistently, results were also observed at 12-h time point (Supplementary Figure 5E and F).

Next, siRNA target PFKFB3 was transfected to ACHN cells. Similarly, PFKFB3 deprivation increased Akt phosphorylation and attenuated rasfonin-dependent autophagic flux (Figures 7c and d).

### Inhibition of PFKFB3 fails to decrease rasfonin-induced apoptosis

In addition to the induction of autophagy, PFKFB3 is actively involved in apoptosis.^39, 40^ Given that API-2 inhibits both PFKFB3 expression and PARP-1 cleavage, we assumed that Akt might positively regulate the rasfonin-dependent apoptosis via the glycolytic pathway. Either PFK-15, 3-PO alone or in combination with API-2 inhibited rasfonin-induced autophagy at the 12-h time point (Supplementary Figure 5E and F). Nevertheless, PFK-15 promoted rasfonin-induced PARP-1 cleavage (Figure 7e,Supplementary Figure 5C), as demonstrated in 3-PO-treated cells (Figure 7f). However, API-2 reduced the PARP-1 cleavage induced by both rasfonin/PFK-15 and rasfonin/3-PO (Figures 7e and f); although the presence of PFKFB3 inhibitors notably increased the PARP-1 cleavage in contrast to rasfonin-/API-2-treated cells (Figures 7e and f). Similar to PFK-15 or 3-PO treatment, PFKFB3 deprivation enhanced PARP-1 cleavage in rasfonin-treated cells (Figure 7g).

### Glycolysis disruption by limiting the glucose uptake suppresses rasfonin-induced apoptosis

Recent studies show that loss of function of PFKFB3 shuts the glucose toward the pentose phosphate pathway (PPP), and renders cell apoptosis susceptible.^24, 41^ In the current study, the inhibition of PFKFB3 promotes rasfonin-induced apotosis maybe through the activation of PPP. To explore this hypothesis, we shut down the whole glucose metabolism by interrupting the glucose uptake.

**Figure 8:**
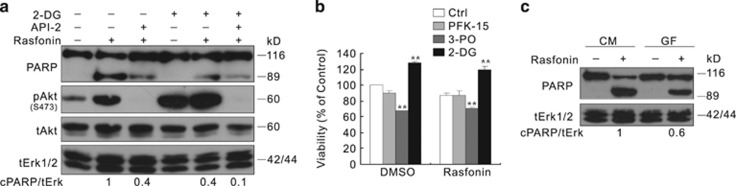
Glucose uptake disruption suppresses rasfonin-induced apoptosis. (**a**) ACHN cells were treated with rasfonin (6 *μ*M) or together with API-2 (2.5 *μ*M) in the presence or absence of 2-DG (5 mM) for 12 h. (**b**) MTS assay was carried out in ACHN cells with rasfonin (6 *μ*M) treatment upon to 24 h in the presence or absence of glycolytic inhibitor (PFK-15: 6 *μ*M; 3-PO: 10 *μ*M; 2-DG 5 mM). (**c**) ACHN cells were treated with rasfonin (6 *μ*M) in completed medium (CM: 10% FBS with glucose) or glucose-free medium (GF) for 12 h. Cell lysates were analyzed by immunoblotting with the antibodies indicated. Relative levels of cPARP-1 were calculated and presented below the blots; tERK1/2 was used as a loading control. Similar experiments repeated twice. The double asterisk denotes statistically different between the marked groups (*P*<0.01)

2-DG is a glucose analog that inhibits glycolysis via its actions on hexokinase, and decreases the G6P level. Here we show that, although 2-DG alone increased autophagic flux, rasfonin together with 2-DG did not promote autophagy (Supplementary Figure 6A). Unlike PFKFB3 deprecation by genetic or pharmacologic methods, the treatment of 2-DG decreased the rasfonin-induced apoptosis (Figure 8a). Furthermore, the combination of 2-DG and API-2 further decreased the rasfonin-induced PARP-1 cleavage (Figure 8a). Additionally, 2-DG was found to block rasfonin-induced cell viability loss at the 12-h time point, neither PFK-15 nor 3-PO showed such an effect (Figure 8b). Furthermore, we transform the cells to glucose-free medium before the indicated treatment. Consistent with the 2-DG treatment results, compare with the results obtained from the full cell medium, the rasfonin-induced autophagy was suppressed under glucose-free condition (Supplementary Figure 6B). The PARP-1 cleavage induced by rasfonin treatment was also decreased in the absence of glucose (Figure 8c).

## Discussion

A new finding in the present study is that Akt positively regulates rasfonin-induced autophagy and apoptosis through the glycolytic pathway. Moreover, rasfonin increases the PFKFB3 expression at mRNA and protein level, which could be suppressed by Akt inhibition. The glycolysis disruption reduces the rasfonin-activated autophagic pathway and PARP-1 cleavage. Interestingly, the isoform-specific roles of Akt in regulating autophagy were revealed in association with cell type and stimulation duration.

As the upstream regulator of mTOR, Akt is typically a suppressor of autophagy.^36, 42^ However, Akt inhibitors failed to stimulate autophagy in rasfonin-treated cells. Indeed, inhibitors of PI3K, an upstream kinase of Akt, either stimulate or inhibit autophagy.^43, 44^ Recently, the class IA PI3K p110-*β* subunit, an upstream regulator of Akt, was reported to positively regulate autophagy.^45^ In the present study, we also observed that Akt1/2 depletion attenuated the induced autophagy in ANCH cells. Moreover, the overexpression of activated Akt stimulated the induced autophagic flux in a time- and Akt isoform-specific manner. These findings indicated that Akt is unlikely to consistently function as an autopahgy suppressor. Therefore, we speculated that Akt might regulate autophagic process in a context-dependent manner.

Akt activation is commonly observed in tumor cells,^18^ and all three isoforms of this kinase were reported to increase cancer cell survival and proliferation.^12^ In the present study, we found that the isoforms differentially regulate autophagy depending on cell type and stimulus duration. Yang *et al.*^17^ observed that the overexpression of constitutively active Akt1 and Akt2 efficiently inhibited the growth of MDA-MB-231 cells. Consistently, overexpression of neither myrAkt1 nor myrAkt2 in ACHN cells stimulates cell growth in the colony growth assay. Moreover, the activated isoforms were unable to improve cellular viability and inhibit PARP-1 cleavage in cells exposed to rasfonin. Consistent with a previous study,^36^ we observed that constitutively active Akt1 reduced mTOR phosphorylation, likely reflecting the increase in apoptotic cell death, as mTOR knockdown increased both Akt phosphorylation and PARP-1 cleavage upon stimulation with rasfonin. In line with an earlier observation,^36^ in which myrAkt1 expression inhibited both basal and induced autophagy, we also observed that rasfonin did not promote autophagy in myrAkt1-transfected cells at the 2-h time point. However, even in ACHN cells, activated Akt regulated autophagy in a time-dependent manner associated with specific Akt isoforms. In addition, we assumed that the amount of glucose in culture medium might affect the regulation of myrAkts on the induced autophagy, as Akt regulates glucose homeostasis with strong isoform specificity.^46^ Akt stimulates aerobic glycolysis in cancer cells, and activated Akt accelerates cell death upon glucose withdrawal.^37^ Indeed, here we show that the pharmacologic or genetic inhibition of Akt reduced *PFKFB3* expression at both mRNA and protein level.

Recently, it was reported that PFKFB3 inhibition suppressed glycolytic flux and tumor growth by rapid induction of apoptosis.^26^ Consistently, we also observed that PFK-15 alone increased PARP-1 cleavage. In the T cells, patients with rheumatoid arthritis and PFKFB3 deficiency restrained activation of autophagy.^24^ Here, we also observed that the loss of PFKFB3 diminished rasfonin-dependent autophagic flux. However, rasfonin stimulated autophagy in FPKFB3-depleted ACHN cells upon longer stimulation concomitant with increased apoptotic cell death. In HCT116 cells, PFKFB3 inhibition induced autophagy as a survival mechanism.^39^ Together with the results obtained in either PFK-15- or 3-PO-treated cells, it is likely that PFKFB3 regulates autophagy depending on time, stimulus, and cell type.

Intracellular glucose is phosphorylated to glucose-6-phosphate (G6P) to enter glycolysis pathway. Alternatively, G6P can proceed through the pentose phosphate pathway (PPP). In U937 cells, glycolysis disruption by the loss of function of PFKFB3 shuts the glucose toward the PPP,^41^ and another study showed that the loss of PFKFB3 enhances the PPP and renders CD4 T-cell apoptosis susceptible.^24^ The glucose analog, 2-DG, has been considered as a promising anticancer agent.^47, 48^ Here, we showed that 2-DG itself could activate autophagy, but decreased the rasfonin-induced autophagy. Interestingly, 2-DG suppressed the rasfonin-activated PARP-1 cleavage. Similarly, results were also observed in the cells treated with glucose-free medium. This part of data indicated that the glycolysis inhibition by loss of function of PFKFB3 may activate the PPP, which enhanced the rasfonin-induced apoptosis. Although the glycolytic pathway fully inhibited by disrupting the glucose uptake, the rasfonin-activated PARP-1 cleavage did not increase any more.

In summary, these data clearly showed that Akt inhibition diminished the rasonin-induced autophagic fluxes, although Akt is considered as a suppressor of autophagy. PFKFB3 could be upregulated by rasfonin and downregulated by Akt inhibition, whereas PFKFB3 deprivation attenuated rasfonin-induced autophagic fluxes. The regulation axis Akt/PFKFB3/autophagy and Akt/PFKFB3/apoptosis had an essential role in ACHN cells when exposed to rasfonin. These results further revealed that the coordination between Akt and the glycolytic pathway has an important role in mediating autophagy and caspase-dependent apoptosis, indicating a new regulatory mechanism for these processes.

## Materials and Methods

### Chemicals and antibodies

3-methyladenine (3-MA, M9281), Rapamycin (R0395), necrostatin-1 (Nec-1, N9037), 2-deoxyglucose (2-DG, D8375), chloroquine diphosphate salt (CQ, C6628), SC66 (SML0261), 1-(4-pyridinyl)-3-(2-quinolinyl)-2-propen-1-one (PFK-15, SML1009), Triciribine hydrate (API-2, T3830), and polyclonal antibodies against LC3 (L7543) were purchased form Sigma-Aldrich (St. Louis, MO, USA). Z-VAD-FMK (FMK001) was purchased from R&D Systems (Minneapolis, MN, USA). Antibodies against PARP-1 (9542), p44/42 MAPK (total-Erk1/2, 9102), phospho-Akt (Ser473, 9271), total Akt (4691), phospho-p70S6Kinase (Thr389,9205), p70S6Kinase (S6K1, 9202), phospho-4E-BP1(Thr37/46) (2855), phospho-mTOR (Ser2448, 2971), and mTOR (2972) were purchased from Cell Signaling Technology (Beverly, MA, USA). Antibodies of Beclin1 (Bec1, sc-11427) and p62 (sc-28359) were acquired from Santa Cruz Biotechnology (Santa Cruz, CA, USA). Total 4E-BP1 (ab32130) and PFKFB3 (ab181861) antibodies were purchased from Abcam (Burlingame, CA, USA). Antibody against actin (TA-09) was obtained from ZhongShanJinQiao Biocompany (Beijing, China). MTS reagent powder (G1111) was acquired from Promega Corporation (Madison, WI, USA). 3-PO was provided by Dr. YQ Huo (Georgia Reagents University, USA).

### Plasmids and siRNAs

The GFP-LC3 plasmid is a kind gift of Dr. Tamotsu Yoshimori (Osaka University, Japan). The myrAkt1 (9008) was obtained from Addgene (Cambridge, MA, USA). The siRNA specific for human MAP LC3*β* (sc-43390), mTOR (sc-35409), PFKFB3 (sc-44011, Akt1 (sc-29195), and Beclin1 (sc-29797) was purchased from Santa Cruz Biotechnology along with the control siRNA (sc-37007).

### Cell culture and immunoblotting analysis

ACHN, 786-O (two human renal cancer cell lines) and HeLa (human cervical carcinoma cell line) were grown in DMEM containing 10% fetal bovine serum (GIBCO, Grand Island, NY, USA), and 1% antibiotics. Cells were grown to 70–80% confluence before adding variety of compounds; the treatments were carried out in completed medium containing 10% serum and gathered the cells at the indicated time point. For transfection, cells grown to 80% confluence were transfected using Lipofectamine 2000 (Invitrogen, Carlsbad, CA, USA) or Attractene (QIAGEN, Hilden, Germany) according to the manufacturer's protocol. After 24–36 h transfection, cells were split and cultured overnight before subjecting to different treatments, immunoblotting or analyzed by confocal microscopy. For siRNA interference, cells were grown to 30% confluence in their respective media without antibiotics and transfected using DharmaFECT (Dharmacon, Lafayette, CO, USA, T2001 or T2002) according to the manufacturer's instructions. Cells were split and cultured overnight before exposure to stimulations after 48 h transfection. Whole-cell lysates were prepared with lysis using Triton X-100/glycerol buffer, containing 50 mM Tris-HCl, pH 7.4, 4 mM EDTA, 2 mM EGTA, and 1 mM dithiothreitol, supplemented with 1% Triton X-100, 1% SDS, and protease inhibitors and then separated on a SDS-PAGE gel (13, 10, or 8% according to the molecular weights for the proteins of interest) and transferred onto PVDF membrane. Immunoblotting was performed using appropriate primary antibodies and horseradish peroxidase-conjugated suitable secondary antibodies, followed by detection with enhanced chemiluminescence (Pierce Chemical, Rockford, IL, USA).

### Cell viability assay (MTS)

Cells were plated in 96-well plates (5000–10 000 cells per well) in 100 *μ*l complete culture medium. After overnight culture, the medium was replaced with complete medium that was either drug-free or contained rasfonin or other chemicals. The cells were cultured for various periods, and cellular viability was determined with CellTiter 96 Aqueous Non-Radioactive Cell Proliferation Assay (Promega).

### Colony growth assay

Cells were seeded at a concentration of 300 cells/ml and cultured for 2 weeks to allow colony growth in the presence or absence of the indicated concentration of rasfonin. Pictures were taken after 4% paraformaldehyde fixation and trypan blue stain, then the numbers of colony were calculated by Image J.

### Flow-cytometry assay

ACHN cells were treated with the indicated compounds, then trypsinized and harvested (keeping all floating cells), washed with PBS buffer, followed by incubation with fluorescein isothiocyanate-labeled annexin V (FITC) and propidium iodide (PI) according to the instructions of an Annexin-V-FITC Apoptosis Detection Kit (Biovision Inc., Milpitas, CA, USA, K101-100) and analyzed by flow cytometry (FACSAria, Becton Dickinson, Franklin Lakes, NJ, USA). Percentages of the cells with annnexin V-positive and PI-negative stainings were considered as apoptotic, whereas PI-positive staining was considered to be necrotic.

### RNA extraction and qRT-PCR analysis

Total cellular RNA was extracted using the TRIzol reagent (Invitrogen, 15596-018) according to the manufacturer's protocol, and its integrity was confirmed by electrophoresis on ethidium bromide-stained 1% agarose gel. Total cellular RNA (1 *μ*g) was reverse transcribed at 37 °C for 15 min in 20 *μ*l of PrimeScriptTM RT reagent Kit (TaKaRa, Dalian, Liaoning, China, DRR037A). Reactions were stopped by heat inactivation for 5 s at 85 °C. Primer sequences used for amplification were as follows:
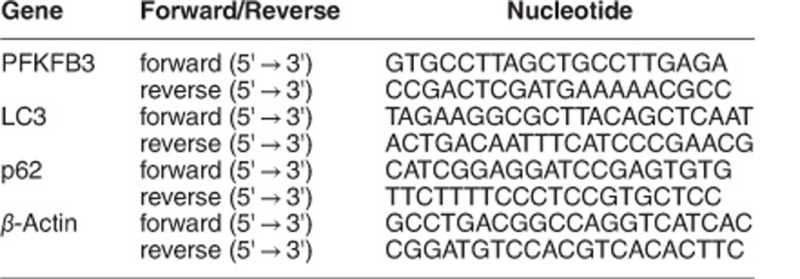


The qRT-PCR (CFX96, Bio-Rad, Hercules, CA, USA) was initiated with a 10-min denaturation at 95 °C in a final volume of 20 *μ*l. The cycle profile was 95 °C (15 s), 60 °C (45 s), and 72 °C (1 min) for up to 40 cycles. Then, data were calculated based on the internal control of *β*-actin.

### Confocal/Fluorescence microscopy

ACHN cells were transiently transfected with GFP-LC3 for 24 h, and split and grown on coverslips overnight before adding rasfonin for the time indicated. Cells were fixed with freshly prepared 4% paraformaldehyde for 12 min at room temperature, and then observed with confocal or fluorescence microscopy.

### Electron microscopy

Electron microscopy was performed as described. Briefly, samples were washed three times with PBS, trypsinized, and collected by centrifuging. The cell pellets were fixed with 4% paraformaldehyde overnight at 4 °C, post-fixed with 1% OsO_4_ in cacodylate buffer for 1 h at room temperature, and dehydrated stepwise with ethanol. The dehydrated pellets were rinsed with propylene oxide for 30 min at RT and then embedded in Spurr resin for sectioning. Images of thin sections were observed under a transmission electron microscope (JEM1230, Japan).

### Statistical analysis

Several X-ray films were analyzed to verify the linear range of the chemiluminescence signals, and the quantifications were carried out using densitometry. Normally distributed data are shown as mean±S.D. and were analyzed using one-way analysis of variance and the Student–Newman–Keuls *post hoc* test. Data are shown as mean±S.D. in graphs.

## Abbreviations

mTOR: mammalian target of rapamycin
Akt: protein kinase B
S6K1: ribosomal protein S6 kinase 1
4E-BP1: eukaryotic initiation factor 4E-binding protein 1
PFKFB3: 6-phosphofructo-2-kinase/fructose-2, 6-bisphosphatase 3
myrAkt: constitutively active Akt
PFK-15: 1-(4-pyridinyl)-3-(2-quinolinyl)-2-propen-1-one
3-PO: 3-(3-pyridimyl)-1-(4-pyridinyl)-2-propen-1-one
PARP-1: poly(ADP-ribose) polymerase-1
2-DG: 2-deoxyglucose
