# Supplementary figures and images for: Akt inhibition attenuates rasfonin-induced autophagy and apoptosis through the glycolytic pathway in renal cancer cells
Source: Cell Death Dis. 2015 Dec 3;6(12):e2005–. doi: 10.1038/cddis.2015.344 (PMC4720880; doi:10.1038/cddis.2015.344)

Suppl. Fig.1

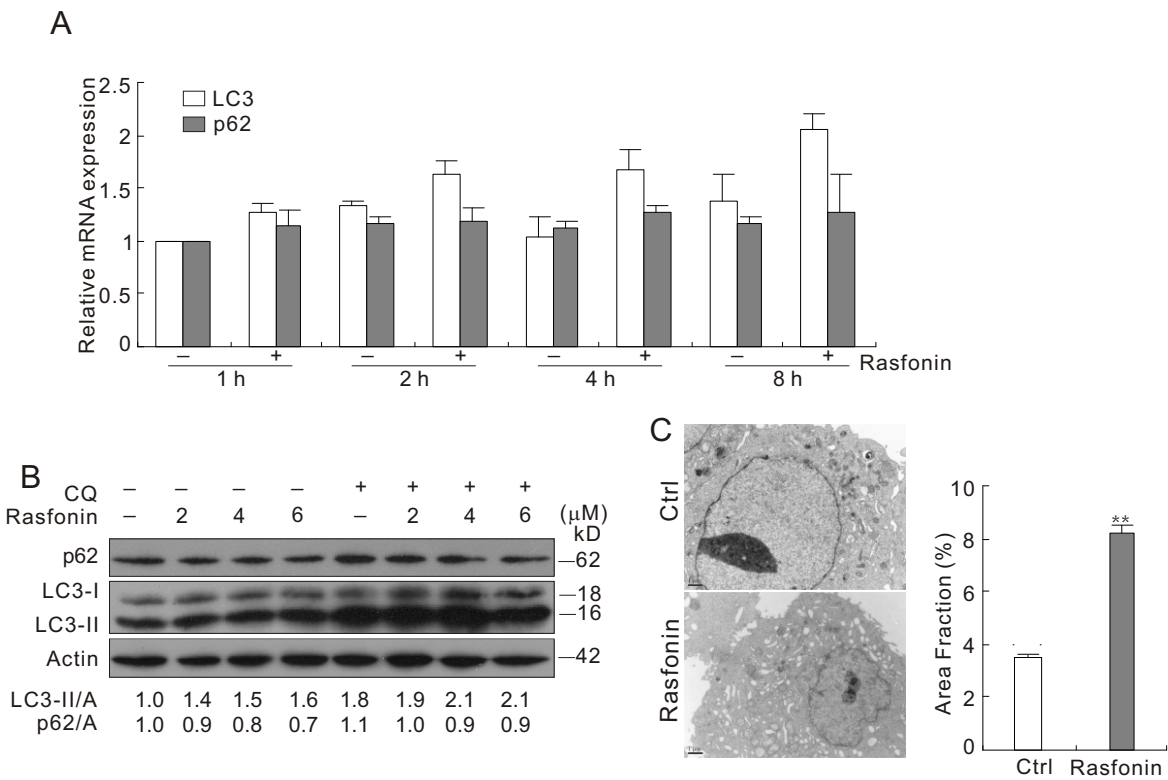

Supplement: Supplementary Figure 1 [file cddis2015344x1.pdf]

Suppl.Fig.2

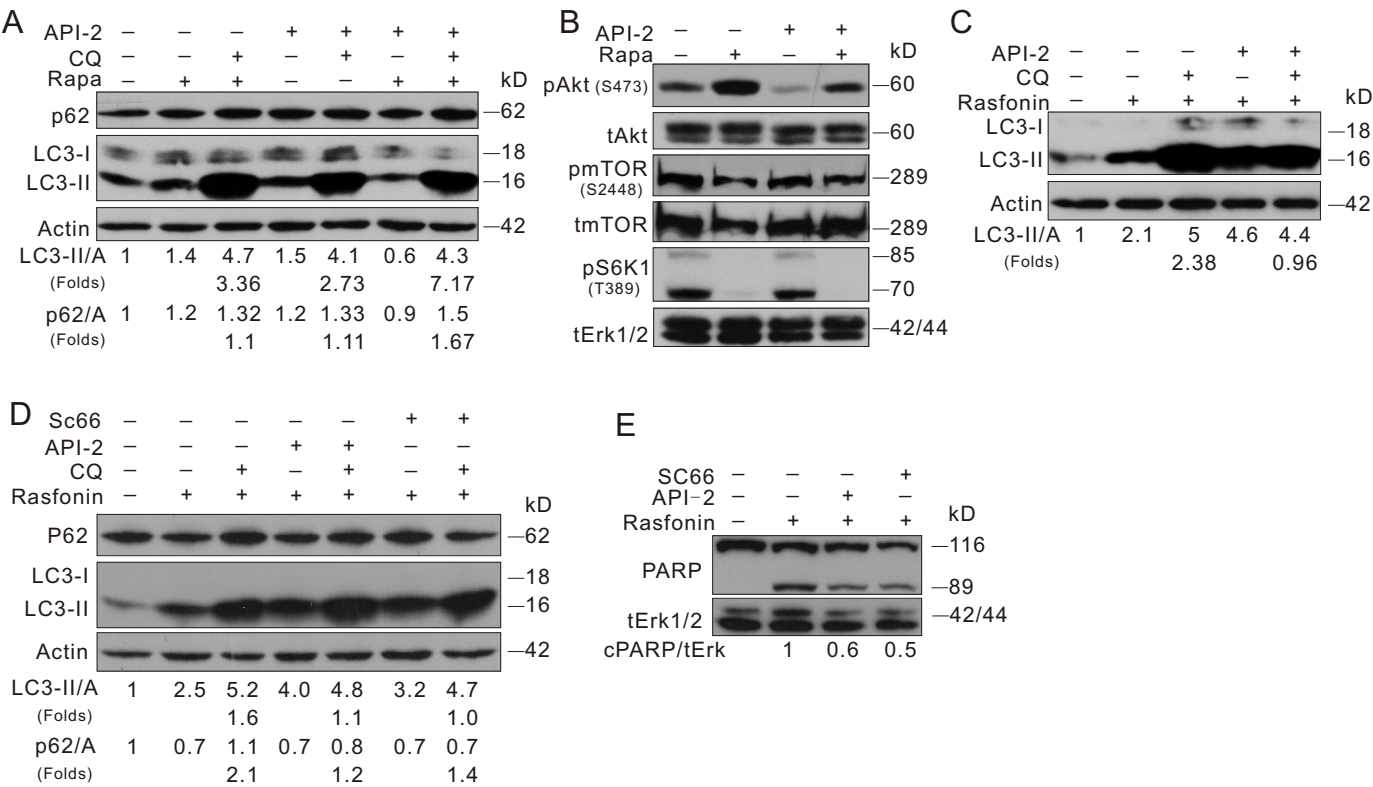

Supplement: Supplementary Figure 2 [file cddis2015344x2.pdf]

Suppl.Fig.3

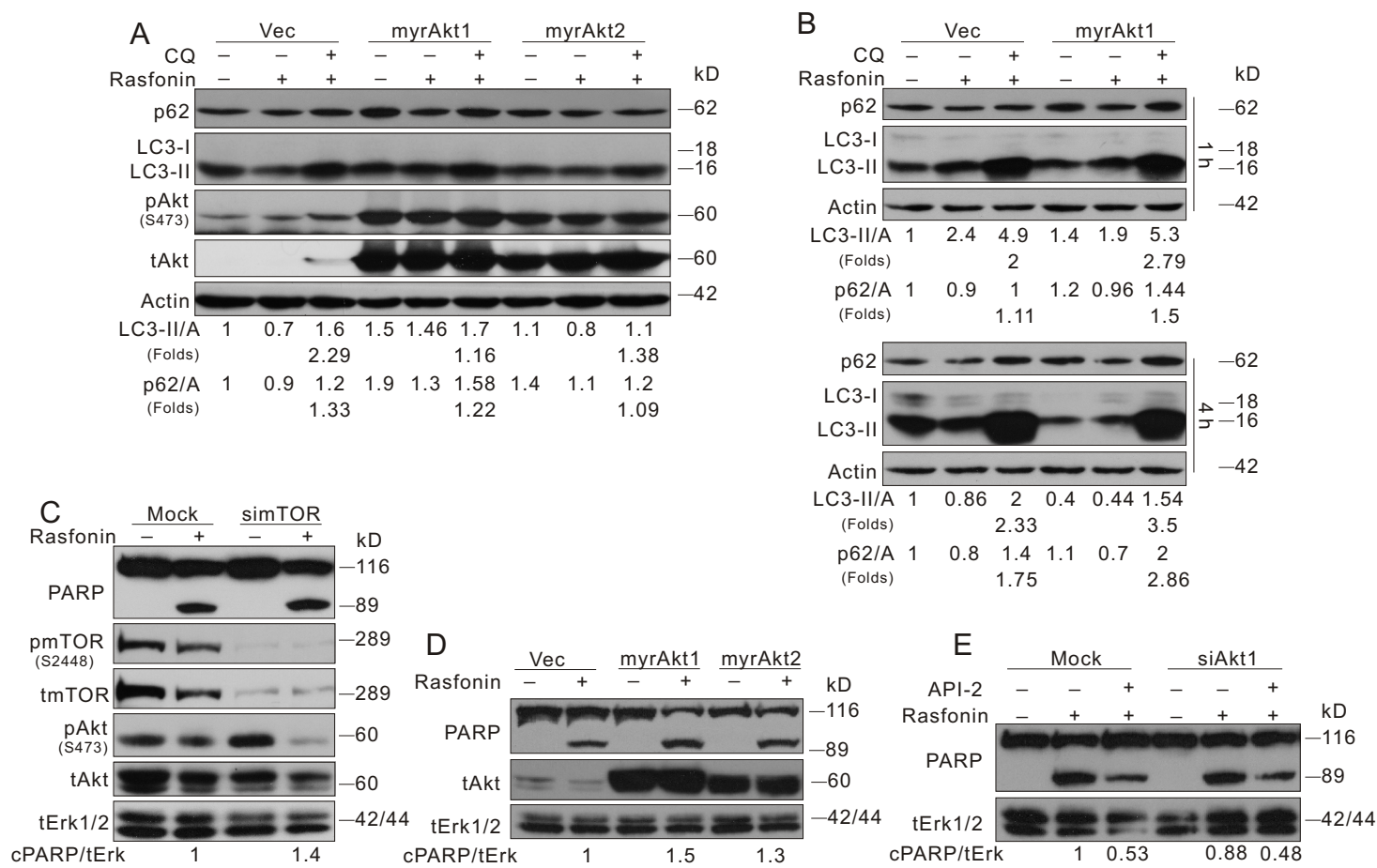

Supplement: Supplementary Figure 3 [file cddis2015344x3.pdf]

Suppl.Fig.4

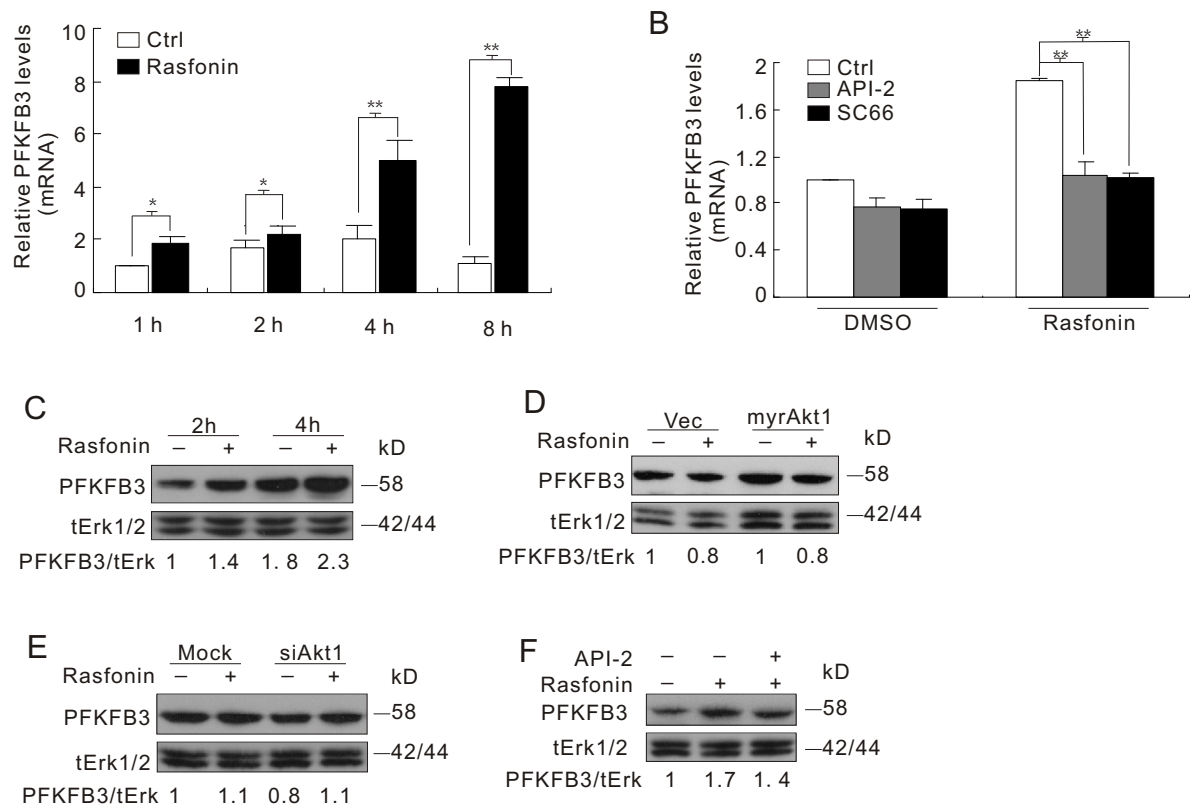

Supplement: Supplementary Figure 4 [file cddis2015344x4.pdf]

Suppl.Fig.5

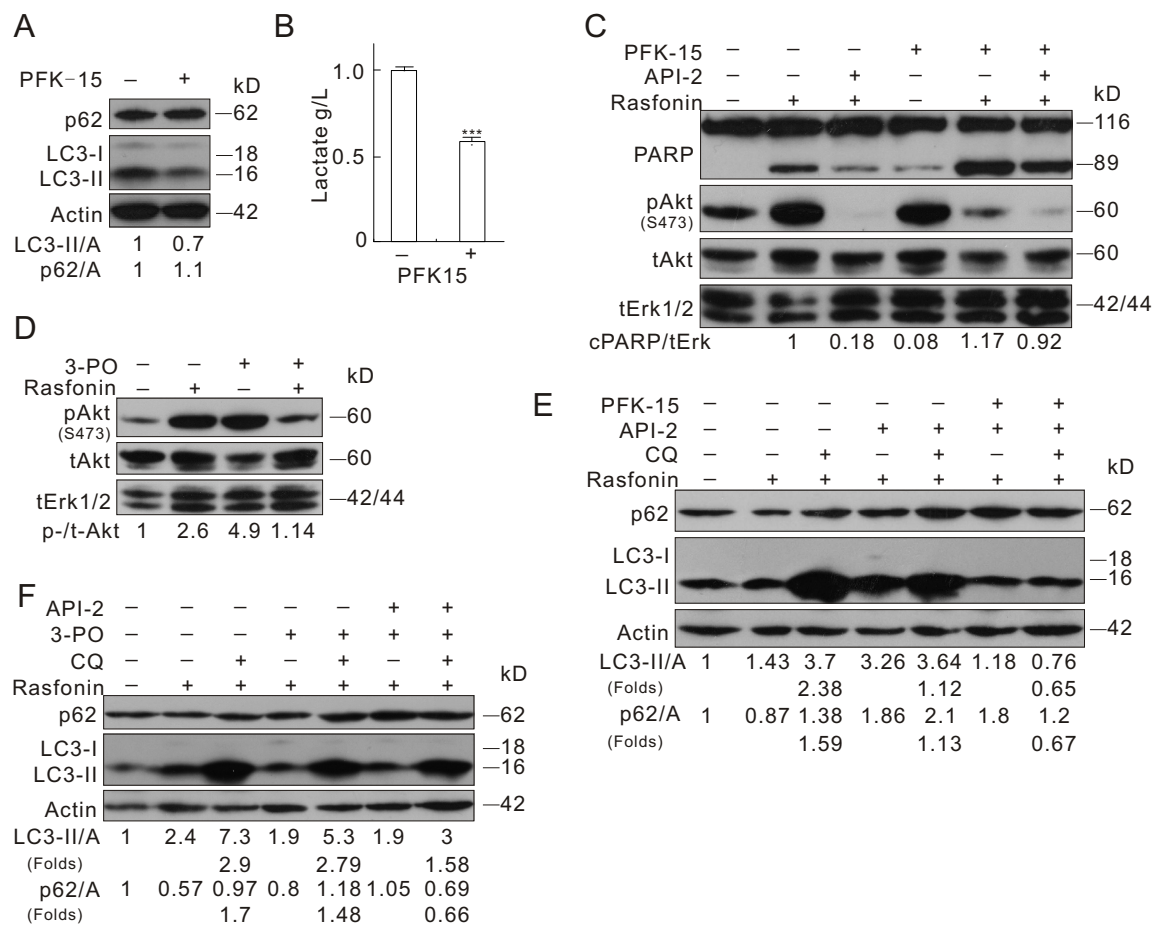

Supplement: Supplementary Figure 5 [file cddis2015344x5.pdf]

Suppl.Fig.6

A

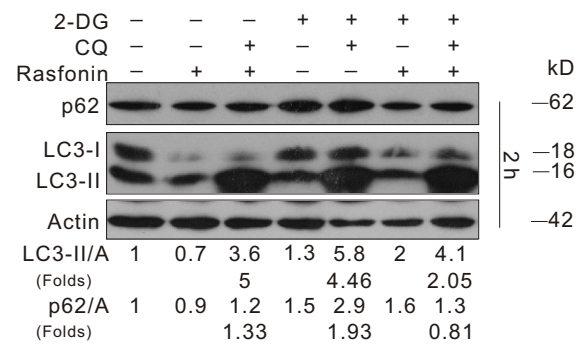

B

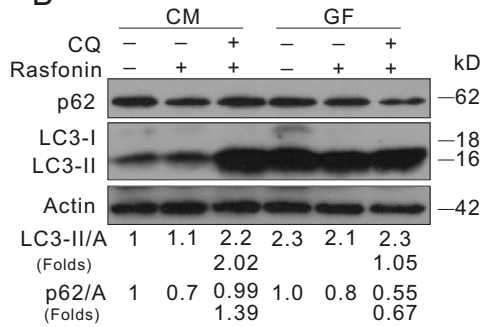

Supplement: Supplementary Figure 6 [file cddis2015344x6.pdf]
