# Supplementary material for: Akt inhibition attenuates rasfonin-induced autophagy and apoptosis through the glycolytic pathway in renal cancer cells
Source: Cell Death Dis. 2015 Dec 3;6(12):e2005–. doi: 10.1038/cddis.2015.344 (PMC4720880; doi:10.1038/cddis.2015.344)
Supplement: Supplementary Figure Legends [file cddis2015344x7.doc]

**Suppl. Figure legends**

**Suppl. Fig. 1. Rasfonin stimulates autophagy.** (**A**) qPCR was performed to detect the mRNA expression of LC3-II and p62 following treatment of rasfonin (6 μM) in ACHN cells. (**B**) 786-O cells were treated with rasfonin (2-6 μM) for 2 h in the presence or absence of CQ (10 μM). Cell lysates were analyzed by immunoblotting with the antibodies indicated. Relative levels of LC3-II, p62 and phosphorylated Akt were calculated and presented below the blots. (**C**) Electron microscopy was performed in 786-O cells following treatment of rasfonin (6 μM) for 1 h. The morphometric analysis of the area fraction between autophagosomes and cytoplasm was calculated by using Photoshop software. The data of the area ratio were non-normally distributed and are presented as the mean of at least 10 cells counted for each group. Similar experiments repeated twice.

**Suppl. Fig. 2. Akt inhibitors suppress rasfonin-induced autophagy and apoptosis.** (**A**, **B** and **C**)ACHN cells were treated with Rapa (50 nM), or API-2 (2.5μM), or rasfonin (6 μM) in the presence or absence of CQ (10 μM) upon to 2 h (**A** and **B**: 2 h; **C**: 1 h). (**D** and **E**) 786-O cells were treated with API-2 (2.5μM), or rasfonin (6 μM), or SC66 (8 μM) with or without CQ (10 μM) for 12 h. Cell lysates were analyzed by immunoblotting with the antibodies indicated. Relative levels of LC3-II and p62 were calculated and presented below the blots. tERK1/2 was used as loading control in panel **B** and **E**. Similar experiments repeated twice.

**Suppl. Fig. 3. The expression status of Akt isoform affects rasfonin-dependent autophagy and PARP-1 cleavage.** HeLa (**A**), ACHN (**B** and **C**), and 786-O (**D** and **E**) cells were transfected with the indicated plasmids (36 h) or siRNA (48 h). Then the cells were treated with rasfonin (6 μM) in the presence or absence of CQ (ACHN: 10 μM; HeLa: 15 μM) upon to 12 h (**A**: 2 h; **C**, **D** and **E**: 12h). Cell lysates were analyzed by immunoblotting with the antibodies indicated. Relative levels of LC3-II, p62 and cPARP-1 were calculated and presented below the blots. tERK1/2 was used as loading control in panel **C**, **D** and **E**. Similar experiments repeated twice.

**Suppl. Fig. 4. Akt inhibition reduces the expression of glycolytic genes *PFKFB3*.** (**A** and **B**) qPCR was performed to detect the mRNA expression of PFKFB3 following treatment of rasfonin (6 μM), or API-2 (2.5 μM), or SC66 (8 μM), upon to 8 h (**B**: 2 h) in ACHN cells. (**C**) ACHN cells were treated with rasfonin (6 μM) for 2 h and 4h. (**D** and **E**) ACHN cells were transfected with either the indicated siRNA (48 h) or plasmids (36 h), and then the cells were treated with rasfonin (6 μM) for 2h. (**F**) ACHN cells were treated with rasfonin (6 μM) in the presence or absence of API-2 (2.5 μM) for 2 h. The lysates of above treatments were subjected to immunoblotting with the indicated antibodies. Relative levels of PFKFB3 were calculated and presented below the blots. tERK1/2 was used as loading control. Similar experiments repeated twice.

**Suppl. Fig. 5. Inhibition of PFKFB3 attenuates the induced autophagy in ACHN cells.** (**A** and **B**) ACHN cells were treated with PFK-15 (6 μM), then the cell lysate (2 h) were collect for immunoblotting and cell medium (24 h) were collect for lactate assay. (**C**, **D**, **E** and **F**) ACHN cells were treated with rasfonin (6 μM) or together with API-2 (2.5 μM), PFK-15 (6 μM), or 3-PO (10 μM) in the presence or absence of CQ (10 μM) upon to 12 h (**D**: 2 h). Cell lysates of above treatments were analyzed by immunoblotting with the antibodies indicated. Relative levels of LC3-II, p62, and cPARP-1 were calculated and presented below the blots. tERK1/2 was used as loading control in panel **C** and **D**. Similar experiments repeated twice.

**Suppl. Fig. 6. Glucose uptake disruption negatively regulates rasfonin-induced autophagic progress. (A)** ACHN cells were treated with rasfonin (6 μM) or together with API-2 (2.5 μM) in the presence or absence of 2-DG (5 mM) for 2 h. (**B**) ACHN cells were treated with rasfonin (6 μM) in completed medium (CM: 10% FBS with glucose) or glucose-free medium (GF) for 2 h. Cell lysates were analyzed by immunoblotting with the antibodies indicated. Relative levels of cPARP-1 were calculated and presented below the blots. Similar experiments repeated twice.
